# CNS Control of Glucose Metabolism: Response to Environmental Challenges

**DOI:** 10.3389/fnins.2013.00020

**Published:** 2013-02-26

**Authors:** Deanna M. Arble, Darleen A. Sandoval

**Affiliations:** ^1^Department of Medicine, University of CincinnatiCincinnati, OH, USA

**Keywords:** arcuate nucleus, brain, glucose metabolism, circadian, high fat diet

## Abstract

Over the last 15 years, considerable work has accumulated to support the role of the CNS in regulating postprandial glucose levels. As discussed in the first section of this review, the CNS receives and integrates information from afferent neurons, circulating hormones, and postprandially generated nutrients to subsequently direct changes in glucose output by the liver and glucose uptake by peripheral tissues. The second major component of this review focuses on the effects of external pressures, including high fat diet and changes to the light:dark cycle on CNS-regulating glucose homeostasis. We also discuss the interaction between these different pressures and how they contribute to the multifaceted mechanisms that we hypothesize contribute to the dysregulation of glucose in type 2 diabetes mellitus (T2DM). We argue that while current peripheral therapies serve to delay the progression of T2DM, generating combined obesity and T2DM therapies targeted at the CNS, the primary site of dysfunction for both diseases, would lead to a more profound impact on the progression of both diseases.

## Introduction

Homeostasis refers to the process of maintaining basic physiological functions despite changes in the internal or external environment. This review will focus specifically on glucose homeostasis, which is the ability to maintain steady blood glucose levels (within 90–120 mg/dl) in spite of nutrient ingestion (feeding vs. fasting), stress (mental or physical), or other environmental challenges. Historically, the role of the CNS in regulating glucose has largely focused on the role of the autonomic nervous system (ANS) in defending against falling glucose levels (glucose counter regulation). However, a large body of work has accumulated over the last 15 years supporting the role of the CNS in regulating postprandial glucose levels. To do this, the CNS must sense and integrate information stemming from the activity of afferent neurons, and postprandial increases in circulating hormones and nutrients. The CNS then responds by increasing autonomic efferent activity to direct changes in glucose output by the liver and glucose uptake by peripheral tissues (Obici et al., 2002b,c, 2003; Lam et al., 2005a,b,c; Pocai et al., 2005a,c; Sandoval et al., 2008a). The first section of this review will focus on the various signals that act within the CNS to regulate glucose homeostasis. The second section of this review will then focus on the external pressures, including high fat diet, changes to the light:dark cycle, as well as the interaction between these different pressures that contribute to the multifaceted mechanisms that we hypothesize contribute to the dysregulation of glucose in type 2 diabetes mellitus (T2DM). Lastly, we argue that while current peripheral therapies serve to delay the progression of T2DM, if we could generate combined obesity and T2DM therapies targeted to the CNS, a primary site of dysfunction for both diseases, the impact on the progression of both diseases could be much more profound.

## CNS Control of Glucose Homeostasis

**Figure 1:**
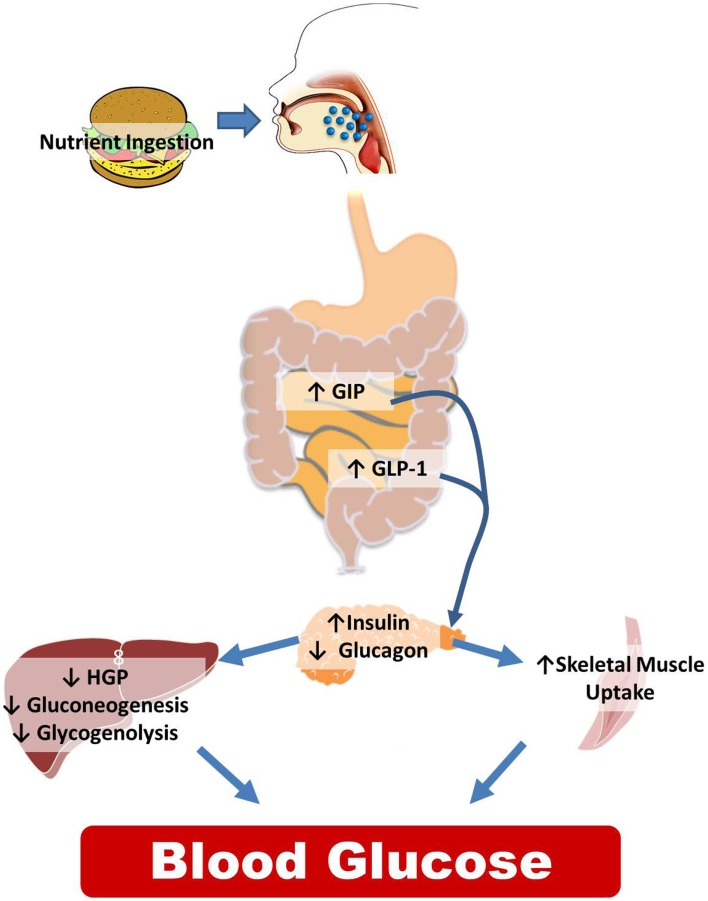
**Peripheral control of glucose homeostasis**. After nutrient ingestion, glucose is absorbed and distributed in equal thirds to the liver, skeletal muscle and adipose tissue, and to remaining tissues including the CNS. The liver both stores (the storage form of glucose is glycogen) and produces glucose that is distributed to the body. Hepatic glucose production is dictated by two metabolic pathways, glycogen lysis (breakdown of glycogen) and gluconeogenesis (production of glucose from non-glucose precursors such as lactate and alanine). Other organs in the body also store glycogen but the amount is miniscule compared to the liver and skeletal muscle. Of the postprandial distribution of glucose, only glucose uptake into the skeletal muscle and adipose tissue is dependent on the increases in insulin released from the pancreas. The role of the intestine in regulating nutrient partitioning is highlighted by the fact that pancreatic β-cell insulin secretion in response to a given glucose load is greater when the glucose is infused into the gut versus when infused intravenously. This is because, incretins, glucose-dependent insulinotropic polypeptide (GIP), and glucagon-like peptide-1 (GLP-1), released from the intestine in a nutrient-dependent manner, act on their receptors located in the β-cell to stimulate the release of insulin. GLP-1 also inhibits glucagon secretion. This change in hormonal milieu, increased insulin, and decreased glucagon levels, have a potent impact on suppressing hepatic glucose production (HGP), and favoring peripheral glucose uptake effectively limiting postprandial glycemic excursions.

While the CNS is integral to normal glucose homeostasis, it is important to recognize the peripheral responses to nutrient ingestion as well. This is reviewed in Figure 1 and illustrates the wide array of physiological signals (glucose, GLP-1, insulin, etc.) that are increased postprandially to regulate the balance between liver glucose production and skeletal muscle glucose uptake with the goal of maintaining steady plasma glucose levels. While there are certainly examples of CNS manipulations that regulate insulin secretion or skeletal muscle uptake, the predominant literature suggests that CNS regulates glucose homeostasis via changes in hepatic glucose production (HGP).

**Figure 2:**
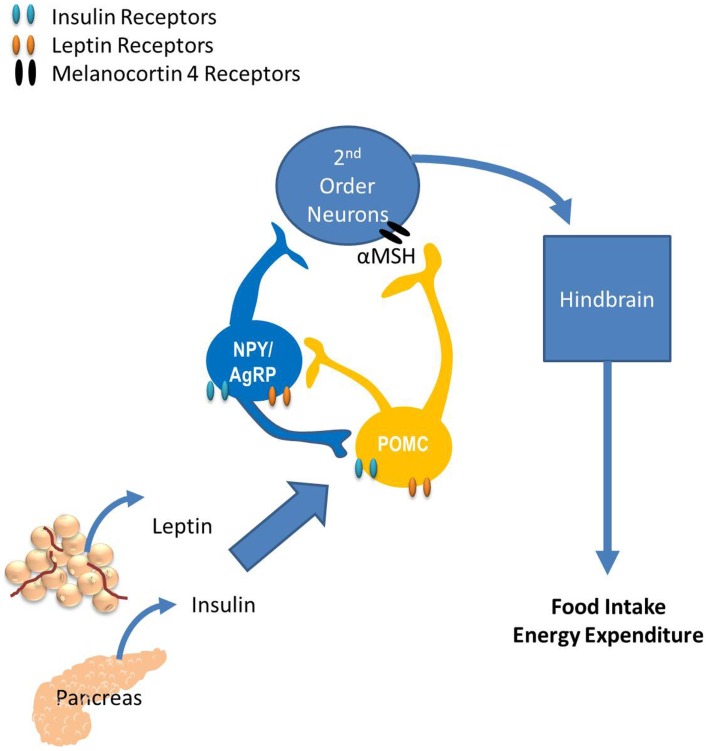
**The role of the arcuate nucleus (ARC) in energy balance**. The ARC has two widely studied neuronal populations that regulate both energy and glucose homeostasis: neurons that express the anabolic peptides, neuropeptide Y (NPY) and Agouti-related peptide (AgRP), as well as the catabolic peptide, proopiomelanocortin (POMC). POMC neurons release α-melanocyte-stimulating hormone (α-MSH) which binds to melanocortin receptors located on second-order neurons within the paraventricular, dorsomedial, and ventromedial nuclei and the lateral hypothalamus. Subsequently, these neurons project to the hindbrain and the periphery, providing a route of communication between the hypothalamus and the periphery via autonomic, neurohumoral, and somatomotor responses. Fasting increases NPY and AgRP expression, consistent with their role as anabolic effectors and feeding increases POMC expression. The response of these circuits to the changing status of stored and ingested nutrients reflects their role in integration of short- and long-term regulation of energy homeostasis.

Much of what we know regarding the neurocircuitry that regulates glucose homeostasis has been extended from the known neurocircuitry regulating energy homeostasis. The paraventricular nucleus (PVN), the ventromedial hypothalamus (VMH), the lateral hypothalamus, and specific neurons within the hindbrain have all been shown to regulate both energy and glucose homeostasis. However, the role of the arcuate nucleus (ARC) on regulating both energy and glucose homeostasis is the most widely studied. Neurons within the ARC that express the anabolic peptides, neuropeptide Y (NPY) and Agouti-related peptide (AgRP), and the catabolic peptide proopiomelanocortin (POMC; Sandoval et al., 2008b) have specific and important roles in regulating energy and glucose homeostasis. Their role in regulating energy homeostasis is reviewed in Figure 2. In this section we discuss the impact of endocrine and nutrient signals that work via the CNS, primarily via ARC neurons to regulate glucose homeostasis.

### Endocrine: Insulin and leptin

As illustrated in Figure 1, insulin is essential for the regulation of glucose homeostasis by acting directly on insulin receptors within the liver and skeletal muscle. Insulin, in addition to the adipokine leptin, circulate in proportion to body fat (Caro et al., 1996; Considine et al., 1996) and thus serve to inform the CNS about the current amount of body fat in order to regulate long-term energy stores. Both circulating leptin and insulin penetrate the blood-brain barrier and bind to their receptors located in several brain regions including in the ARC (Van Houten et al., 1979, 1980; Tartaglia et al., 1995). While insulin acts centrally to regulate energy homeostasis, it acts both centrally and peripherally to regulate glucose homeostasis. Indeed, mice with genetic deletion of neuronal insulin receptors are insulin resistant (Bruning et al., 2000). Even more specific, activation of insulin receptors within the ARC have a very potent effect on suppressing HGP by reducing both gluconeogenesis and glycogenolysis (Obici et al., 2002c) and this effect is independent of its effect on regulating food intake (Obici et al., 2002a).

Given the importance of insulin receptors on POMC and NPY/AgRP neurons in energy homeostasis (Schwartz et al., 2000), it is logical to propose that insulin receptors located on these neurons also have a role in regulating glucose homeostasis. However, genetic deletion (Konner et al., 2007) or reactivation (Lin et al., 2010) of insulin receptors specifically on POMC neurons does not alter hepatic insulin sensitivity in a way that can be separated from its impact on energy expenditure. These data are consistent with data demonstrating that melanocortin receptor antagonists delivered into the third cerebral ventricle do not block the ability of central insulin to reduce HGP (Obici et al., 2001). Conversely, when insulin receptors are selectively removed or reactivated specifically within AgRP neurons, hepatic insulin sensitivity is respectively impaired or improved (Konner et al., 2007). Thus, insulin receptor signaling on “anabolic,” but not “catabolic,” neurons improves glucose homeostasis. AgRP neuronal activity is inhibited by insulin suggesting that the inhibitory action of insulin on AgRP neurons is responsible for insulin-induced improvements in HGP. Thus, insulin resistance within AgRP neurons could remove a major inhibitory signal to these neurons ultimately contributing to impaired hepatic insulin sensitivity.

When administered centrally, leptin also regulates glucose homeostasis but in a slightly more complicated manner; via the ARC (German et al., 2009) leptin decreases glycogenolysis, but increases gluconeogenesis thus resulting in no net change in HGP (Liu et al., 1998; Pocai et al., 2005b). Like insulin, leptin action on POMC and AgRP neurons is integral in its influence on glucose homeostasis. The hepatic insulin resistance seen in leptin receptor null mice is normalized by genetic reactivation of leptin receptors specifically in POMC neurons in a weight-independent fashion (Berglund et al., 2012). However, pharmacological antagonism of melanocortin receptors only inhibits leptin-induced increases in gluconeogenesis with no effect on glycogenolysis (Gutierrez-Juarez et al., 2004), suggesting that leptin’s action on regulating hepatic glucose fluxes is only partly mediated via the melanocortin system. Given the impact of insulin signaling on AgRP neurons, it would be interesting to understand the coordinated impact of leptin receptors on both sets of neurons.

Being an integrated system, it is not surprising that insulin and leptin signaling converge on POMC neurons to regulate glucose homeostasis. While deletion of leptin receptors from POMC neurons results in obesity, this effect is partially prevented when both leptin and insulin receptors are deleted from these neurons yet, these double-knockout mice have a greater impairment in insulin tolerance and hepatic insulin resistance compared to control animals (Hill et al., 2010). However, insulin and leptin receptors are not co-localized to the same individual POMC neuron (Williams et al., 2010). Instead, some POMC neurons express the leptin receptor and some express the insulin receptor, suggesting that these signaling pathways interact between, rather than within, POMC neurons.

Insulin and leptin receptors are also located within the VMH. Leptin signaling initiates its own negative feedback loop via a protein called suppressor of cytokine signaling (SOCS3). Genetic inactivation of SOCS3 specifically within steroidogenic factor-1 (SF-1) neurons that are primarily expressed within the VMH, reduces both food intake and energy expenditure leading to no differences in body weight compared to control animals, but also normalizes high fat diet-induced hyperinsulinemia (Zhang et al., 2008a). Further, insulin receptors genetically deleted from SF-1 neurons results in a mouse that is resistant to diet-induced obesity and diet-induced leptin resistance (Klöckener et al., 2011) and has reduced hyperinsulinemia with improved systemic glucose and insulin tolerance. Thus, both insulin and leptin signaling within the VMH are important for regulating glucose homeostasis.

To summarize, both insulin and leptin act on POMC, AgRP, and VMH neurons to regulate glucose homeostasis but in a complicated manner. Alone, insulin’s inhibitory action on AgRP, but not POMC, neurons is necessary for the regulation of glucose homeostasis. Insulin and leptin both act individually on SF-1 neurons, but have additive action on POMC neurons to regulate glucose homeostasis. These data highlight the wide array of neurocircuitry that mediate insulin and leptin action, and bring to light the possibility that insulin and/or leptin resistance that occurs with the progression toward T2DM at any of these circuits could significantly impact glucose homeostasis.

### Nutrient signaling

In addition to hormonal signals like leptin and insulin, the CNS is responsive to nutrient derived signals. Both glucose (Levin, 2006) and lipids (Wang et al., 2006) directly regulate neuronal activity within the ARC, VMH, and nucleus of the solitary tract by either increasing or decreasing neuronal depolarization. Further, direct administration of nutrients or nutrient metabolites such as glucose, lactate, long-chain fatty acids, or leucine into the ARC all suppress HGP (Obici et al., 2002c; Lam et al., 2005b; Su et al., 2012). All of these nutrient signals converge on various signaling pathways to modulate neuronal activity and, consequently, physiology.

The energy currency for all cells when nutrients are metabolized is adenosine triphosphate (ATP). Some membrane potassium channel activity (opening vs. closing of the channel) is directly regulated by ATP levels (Parton et al., 2007) and this is used as an important signaling mechanism. One of the most widely characterized ATP-sensitive potassium (K_ATP_) channels is found in pancreatic β-cells where glucose metabolism generates ATP leading to K_ATP_ channel depolarization and eventually insulin exocytosis. Interestingly, neuronal activity in certain neurons within the ARC, VMH, and NTS is also regulated by K_ATP_ channel depolarization (Levin et al., 2004). Within the ARC, pharmacologically depolarizing K_ATP_ channels blocks the ability of glucose, fatty acids, insulin, or leucine to suppress HGP in rodents (Obici et al., 2002c; Lam et al., 2005b; Su et al., 2012). Genetic disruption of K_ATP_ channels specifically in POMC neurons impairs glucose-sensing but does not alter the response of these neurons to leptin, and while these mice have normal body weights, they have impaired glucose tolerance (Parton et al., 2007). Surprisingly, mice with dysfunctional K_ATP_ channels within melanin concentrating hormone neurons, orexigenic neurons within the lateral hypothalamus, have normal body weights but impaired glucose tolerance (Kong et al., 2010). Thus, hypothalamic K_ATP_ channels within the ARC and lateral hypothalamus are a common mechanism through which different types of nutrients (and insulin but not leptin) and neurons regulate glucose homeostasis.

Two other intracellular signaling pathways that have important roles in monitoring cellular nutrient status also play a role in regulating hypothalamic nutrient sensing and consequently whole-body energy and glucose homeostasis. AMP-activated protein kinase (AMPK) is activated by low ATP levels, and mammalian target of rapamycin (mTOR) is activated by a surplus in ATP. Increases in hypothalamic AMPK drives increases in food intake (Minokoshi et al., 2004), and shifts glucose from the liver to the muscle by increasing HGP and muscle glycogen synthesis (Perrin et al., 2004). Genetic disruption of AMPK specifically in POMC or AgRP neurons causes obesity or resistance to obesity, respectively (Claret et al., 2007), while the neurons themselves maintain the ability to respond to insulin and leptin but not glucose. This alteration in body weight confounds the interpretation of this signaling pathway on regulating glucose homeostasis as changes in body weight independently regulate glucose homeostasis. However, the data do suggest that these neurons have an important impact on body weight regulation but that leptin and insulin act on these neurons via non-AMPK dependent pathways (Claret et al., 2007).

Mammalian target of rapamycin is a ubiquitously expressed kinase involved in determining cell growth. Consequently, mTOR expression within the hypothalamus is increased after feeding and decreased after fasting (Cota et al., 2006). Leucine administered into the CNS suppresses food intake via activation of the hypothalamic mTOR pathway (Cota et al., 2006), yet the ability of leucine to suppress HGP is instead mediated by K_ATP_ channel rather than mTOR activation (Su et al., 2012). Although presumably independent of leucine, hypothalamic mTOR is still important for regulating glucose homeostasis. Chronic activation of the TOR pathway within the ARC reduces diet-induced obesity (Blouet et al., 2008), but conversely increases HGP at time points where there is minimal changes in body weight (Ono et al., 2008). Thus, energy surplus sensed by the hypothalamus activates the mTOR pathway and subsequently leads to a reduction in body weight but an increase in hepatic glucose production (HGP). These divergent effects may depend on the duration of activation and may represent a mechanism to shift fuels to skeletal muscle. Furthermore these data demonstrate that the regulation of body weight can be dissociated from the regulation of glucose homeostasis.

Thus far we have discussed how hypothalamic neurons sense overall energy status by monitoring ATP levels. However, molecular mechanisms exist that allow for sensing of specific types of fuel. For example, various fatty acids serve as ligands for peroxisome proliferator activated receptors (PPAR). One specific isoform is PPARγ, a nuclear transcription factor which functions to direct lipid storage to adipose tissue and away from the liver and skeletal muscle as fat accumulation in these organs would impair insulin sensitivity. Clinically, PPARγ agonists (thiazolidinediones) effectively treat hyperglycemia in T2DM but also cause weight gain. Interestingly, PPARγ is expressed in the CNS, and in rats, both CNS administration of a PPARγ agonist, or viral induction of hypothalamic PPARγ expression (Ryan et al., 2011) increase body weight suggesting that the clinical side effect is via the CNS. On the other hand, mice that lack PPARγ specifically within the CNS are resistant to dietary-induced obesity and do not increase feeding or show improvements in hepatic insulin sensitivity when administered PPARγ agonists (Lu et al., 2011). Thus, CNS PPARγ activation has a potential role in the regulation of adiposity, is responsible for the clinical increase in body weight, and at least partially regulates the improvements in glucose homeostasis seen with thiazolidinediones.

There is some evidence that the neuronal control of the glucose homeostasis could be aided by specialized glia cells called tanycytes. The tanycyte cell body lines the third ventricle of the hypothalamus and a subset of tanycytes (α1 and α2) project to the ARC and ventromedial hypothalamic nucleus (VMH). It is hypothesized that glucose levels in the CSF can be detected by the tanycyte and this information is used to maintain glucose levels (Rodríguez et al., 2005). The process by which this happens is proposed to be similar to pancreatic β cells for which glucose-sensing is key to the function of the cell. For example, tanycytes express glucose transporters (Peruzzo et al., 2000; García et al., 2003) and use the metabolism of glucose to generate ATP and subsequently modulate cellular depolarization (Dale, 2011; Frayling et al., 2011). One could hypothesize that if these cells are key to regulating glucose homeostasis then they may be upstream of hypothalamic nuclei known to regulate HGP and/or glucose uptake. While these cells clearly sense glucose and it is interesting to propose that they play a role in regulating glucose homeostasis, this is entirely speculative.

### Hypothalamic MC4R

Thus far, we have discussed several pieces of data demonstrating that the ARC POMC and/or NPY/AgRP neurons are important for CNS-induced regulation of glucose homeostasis. POMC neurons that project to the PVN can release GABA, an inhibitory neurotransmitter, or α-MSH, an agonist for PVN-expressed MC4R. An interesting question is whether MC4R are downstream of POMC activation in regulation of glucose homeostasis. MC4R alone are clearly important for glucose homeostasis as demonstrated by the fact that MC4R knockout mice are hyperinsulinemic prior to the onset of obesity (Huszar et al., 1997) and have hepatic and peripheral insulin resistance (Rossi et al., 2011). In addition, chronic ICV administration of an MC4R agonist improves, while an MC3/MC4R antagonist impairs, hepatic, and skeletal muscle insulin sensitivity in a weight-independent fashion (Obici et al., 2001).

One example that supports MC4R are downstream of POMC activation is serotonin (5HT). 5HT is synthesized in the dorsal raphe nucleus of the midbrain and is released into the hypothalamus, including the ARC (Kiss et al., 1984) where it binds to type 2C receptors (5HT_2c_; Heisler et al., 2002). 5HT_2C_ null mice have increased body weight and hepatic insulin resistance and reactivation of 5HT_2C_ receptors specifically in POMC neurons normalizes hepatic insulin sensitivity (Xu et al., 2010) suggesting that these specific receptors are sufficient to regulate energy and glucose homeostasis. Although deletion of both leptin and the 5HT_2C_ receptors have additive and weight-independent impairments in fasting glucose and glucose tolerance (Wade et al., 2008), the data indicate that this is mediated via separate populations of POMC neurons (Sohn et al., 2011). Interestingly, MC4R null mice do not respond to pharmacological activation of 5HT signaling, and MC4R reactivation specifically within the PVN and amygdala restores the effect (Xu et al., 2010) suggesting that the effect of POMC activation is via MC4R. While it is true that MC4R do not seem to be downstream of insulin action within the CNS to regulate glucose homeostasis (Obici et al., 2001), MC4R are downstream of 5HT activation and at least partially downstream of leptin.

### Hindbrain MC4R

Proopiomelanocortin neurons and MC4R are also expressed within the hindbrain, and recent data demonstrate their importance in regulating glucose homeostasis (Rossi et al., 2011). Reactivation of MC4R specifically in cholinergic preganglionic sympathetic and parasympathetic neurons located in the dorsal motor nucleus of the vagus (DMV) attenuates obesity and hepatic, but not skeletal muscle, insulin resistance seen in MC4R null mice (Rossi et al., 2011). However, when MC4R were only reactivated in parasympathetic neurons (afferent neurons located in the nodose ganglia) the only effect was attenuation of hyperinsulinemia (Rossi et al., 2011). Together, these data suggest that MC4R signaling within the parasympathetic nervous system is important in specifically regulating insulin secretion but not overall glucose homeostasis. Lastly, while MC4R located within the hypothalamus and hind brain regulate glucose homeostasis, it remains unknown if these systems are integrated in glucoregulation.

### Gut-brain-liver axis

The gut is an important barrier to exogenous nutrients entering the circulation. When exposed to nutrients, not only is there an increase in neuronal activity in vagal afferents that project directly to hindbrain sympathetic neurons (DiRocco and Grill, 1979), but there is also an intestinal release of several hormones, neuropeptides, and neurotransmitters that relay changes in nutritional status to the rest of the body. Evidence suggests that two particular gut peptides secreted postprandially, cholecystokinin (CCK) and glucagon-like peptide-1 (GLP-1), have action via vagal afferents to regulate glucose homeostasis forming a gut to brain to liver axis.

Cholecystokinin is secreted from the proximal intestine in response to lipid ingestion and functions to regulate satiety, digestion, and gastric emptying. CCK receptors are found on vagal afferent axons where its action on satiety is mediated (Strader and Woods, 2005). Recent work suggests that these receptors also regulate HGP. Infusion of a small amount of lipids sufficient to increase CCK but with limited impact on plasma triglycerides (Wang et al., 2008) or a direct infusion of CCK (Cheung et al., 2009) into the upper intestine of rats suppresses HGP. *N*-methyl-d-aspartate channels in the dorsal vagal complex of the hindbrain (Lam et al., 2010) play an important role in relaying these signals from the gut to the hindbrain and eventually back to the liver to regulate HGP.

Glucagon-like peptide-1 (GLP-1) is a gut hormone secreted postprandially from the distal small intestine. Initially studied for its important role in insulin secretion and thus glucose homeostasis, GLP-1 is now known to have a wide array of physiological effects including lipid homeostasis and cardiovascular function (Drucker, 2006). GLP-1 is also produced within the hindbrain where it is thought to regulate food intake and visceral illness (Barrera et al., 2011).

Peripherally secreted GLP-1 likely has limited endocrine action as it has a short circulating half-life due to rapid degradation (Kieffer and Habener, 1999). However, GLP-1 receptors are located on afferent neurons that innervate the hepatic portal vein (Vahl et al., 2007) and on enteric neurons within the gastrointestinal tract (Amato et al., 2010). These afferent neurons within the portal vein are important for regulating glucose tolerance (Vahl et al., 2007) and meal patterns (Ruttimann et al., 2009). In addition, both intravenous GLP-1 in humans (Prigeon et al., 2003), and GLP-1 administered directly into the ARC in rats (Sandoval et al., 2008a) decrease HGP. Furthermore, intracerebroventricular administration of GLP-1 in rats also stimulates insulin secretion (Knauf et al., 2005; Sandoval et al., 2008a). We postulate that GLP-1 secreted by the intestine activates afferent neurons which then activate the CNS to in turn suppress HGP. Together these data suggest a coordinated response of intestinal and portal vein afferent neurons in response to postprandially secreted CCK and GLP-1 leading to efferent activity to the liver via the CNS and thus the regulation of glucose production by a gut to brain to liver axis.

### Neuroanatomy of the CNS to peripheral organs

The ANS plays an integral role in regulating peripheral glucose homeostasis at the liver, muscle, and even indirectly by regulating pancreatic hormone secretions. The ANS consists of the parasympathetic and sympathetic branches. The parasympathetic branch is activated postprandially; its effects being mediated primarily by the vagus nerve which innervates organs of the visceral cavity and thus, impacts nutrient digestion, absorption, and metabolism. Several studies, primarily from one group, have demonstrated that the hepatic branch of the vagus is essential for the ability of insulin and nutrients infused directly into the CNS to suppress HGP (Lam et al., 2005a; Pocai et al., 2005a,c; Cheung et al., 2009). Acetylcholine is the main neurotransmitter released from vagal efferent neurons, and given the previously described role of the vagus, it is surprising that neither specific over-expression or reduction of expression of hepatic M3 receptors impacts normal regulation of HGP in mice (Li et al., 2009). The authors postulated that other neurotransmitters co-released with acetylcholine from vagal terminals (e.g., vasoactive intestinal polypeptide, gastrin-releasing peptide, pituitary adenylate cyclase-activated peptide, and nitric oxide) are responsible for linking the CNS to regulation of HGP. Indeed, in isolated hepatocytes, nitric oxide has potent inhibitory effects on hepatic gluconeogenesis (Horton et al., 1994), but nothing is known about its role *in vivo*.

Conversely, the sympathetic branch is activated in times of physiological stress including exercise and hypoglycemia. Its primary neurotransmitters, norepinephrine, and epinephrine, act on the liver to increase glucose production and on skeletal muscle to decrease glucose uptake in order to maintain glucose levels. On the other hand, the sympathetic nervous system plays a prominent role in hindbrain MC4R-induced suppression of HGP (Rossi et al., 2011). Like most organs regulated by the ANS, the balance between parasympathetic and sympathetic activity to the liver is likely important. It is possible that with the various experimental models there is a shift in the importance of one of the limbs of the ANS. Conversely, given the importance of HGP to physiological function, signals processed by the ANS could lead to a hepatic outcome that results from the integration of the signals involved in reflecting nutrient status.

## Environmental Challenges to the CNS Control of Glucose Metabolism

In the preceding section we discussed several genetic and pharmacological manipulations that impair glucose homeostasis. This basis allows for the exploration of CNS mechanisms that regulate glucose homeostasis. However, chronic environmental challenges such as consumption of high fat diets and a non-stop 24 h society both place a homeostatic challenge on the CNS. This leads to a key question of whether these circuits that regulate glucose homeostasis become dysfunctional in the face of these chronic environmental challenges and whether this dysfunction contributes to the onset or progression of T2DM. In the following section, we discuss how high fat diets and circadian rhythms can influence the CNS control of glucose metabolism.

### High fat diets

The consumption of a diet high in fat leads to defective hormonal and nutrient sensing within peripheral organs. This in turn contributes to metabolic dysregulation, including insulin resistance that occurs as a hallmark of T2DM. However, the CNS is not immune to the effects of high fat diet. The reduced hormonal and nutrient sensing that occurs within the CNS impairs CNS-induced regulation of both energy and glucose homeostasis. Specifically, high fat diets rapidly and independent of adiposity reduce the ability of ICV insulin, leptin, and fatty acids to reduce food intake and HGP (Arase et al., 1988; El-Haschimi et al., 2000; Wang et al., 2001; Morgan et al., 2004; Woods et al., 2004).

The mechanism for this effect is unknown but there is evidence of impaired transport of both leptin and insulin across the blood-brain barrier (Stein et al., 1987; Israel et al., 1993; Banks et al., 1999; Banks, 2008). Similarly, both leptin (Banks et al., 1996) and insulin (Stein et al., 1983; Israel et al., 1993) receptor expression is decreased within the CNS in obesity. However, impairments are also found within the respective signaling pathways (El-Haschimi et al., 2000; Munzberg et al., 2004). Activation of hypothalamic inflammatory pathways, which occurs on a high fat diet, also causes insulin and leptin resistance (Zhang et al., 2008b; Ozcan et al., 2009) suggesting a linkage between high fat diets and intracellular events that block insulin and/or leptin signaling. Impairments in neuronal circuitry downstream of the ARC are also found as MC4R are resistance to pharmacological activation in animals exposed to a high fat diet (Clegg et al., 2003). One intriguing aspect of CNS leptin signaling is that while the anorectic action of leptin is blunted on a high fat diet, central infusion of leptin still lowers gluconeogenesis and overall HGP (Morton et al., 2005; Pocai et al., 2005b) independently of insulin levels (German et al., 2011).

Expression of a specific component of the K_ATP_ channel (Kir_6.2_ subunit) is reduced in the ventromedial and dorsomedial hypothalamus in pre-diabetic compared to non-diabetic zucker rats (Gyte et al., 2007) highlighting that hypothalamic nutrient sensing is also dysfunctional in obesity and could contribute to obesity-induced T2DM. However, more research is needed to determine what specific neurocircuits are defective in obesity and/or with high fat diet exposure. A better understanding these impairments could help elucidate the underlying etiology of obesity and T2DM.

### Circadian rhythms

Many organisms have evolved to predict daily environmental changes and incorporate 24 h rhythms within their physiology. These daily rhythms are known as circadian (from the Latin *circa* meaning “around” and *diem* meaning “a day”) and encompass a broad range of behavioral and physiological characteristics from the sleep-wake cycle to hormonal fluctuations. Arguably, synching metabolic rhythms to the light:dark cycle is greatly beneficial to an organism. This synchronization allows for optimal energy balance tailored to the organisms’ active state as well as an increase in activity and the recruitment of various meal-related hormones (e.g., insulin) prior to a meal.

**Figure 3:**
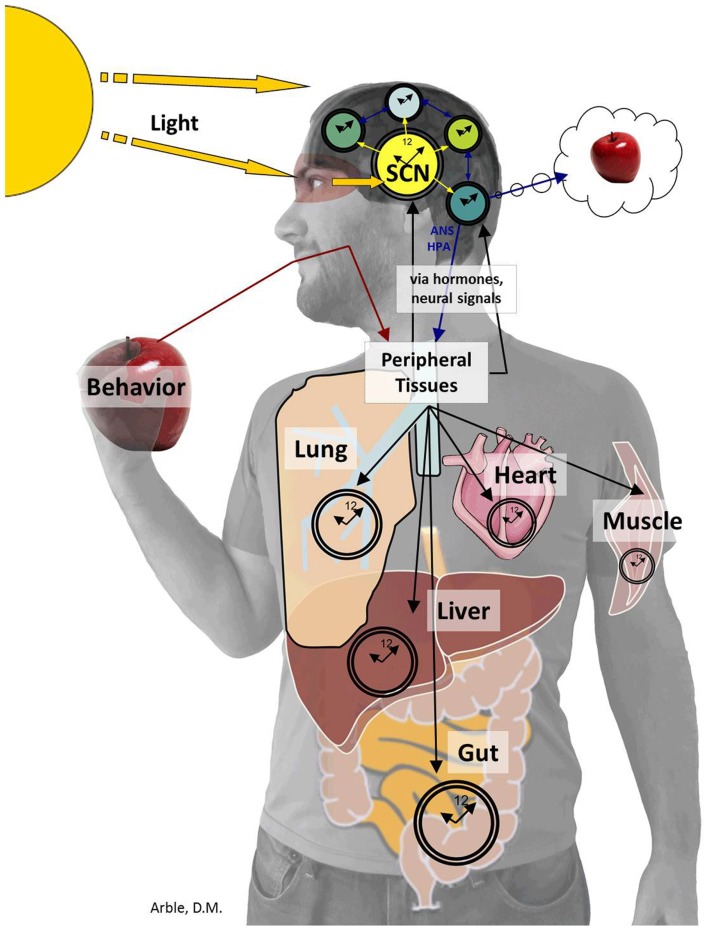
**The SCN sends timing information to the brain and periphery**. The SCN derives “time of day” information from environmental light cues received by specialized cells within the retina (Panda et al., 2002b) and transferred to the SCN by the retinohypothalamic tract. The SCN then conveys timing information to the brain including the PVN, ARC, subparaventricular zone (SPZ), MPOA, intergeniculate leaflet (IGL), and paraventricular nucleus of the thalamus (Kalsbeek et al., 2004, 2008; Froy, 2010; Stenvers et al., 2012). The SCN also provides timing cues to peripheral tissues via the autonomic nervous system and hormones, which together organize complex behaviors from the sleep/wake to the feeding/fasting rhythm.

Just as the ARC is central to regulating postprandial glucose levels, the bilaterally paired, suprachiasmatic nuclei (SCN), located in the anterior hypothalamus, orchestrates circadian rhythms. The SCN derives “time of day” information from environmental light cues and relays this timing information to other areas of the CNS and periphery as depicted in Figure 3. Determining the exact role of the SCN brain clock on overall glucose metabolism is complex. While the SCN is the master oscillator of all rhythms, a number of experiments have shown that altering rhythms downstream of the SCN, such as the rhythm of peripheral tissue circadian genes (Panda et al., 2002a; Kornmann et al., 2007; Lamia et al., 2008; Marcheva et al., 2010; Zhang et al., 2010) or meal timing (Arble et al., 2010) can affect glucose metabolism. For this review, we will focus on the aspects of circadian control of glucose metabolism that have demonstrated to be regulated more by the SCN master clock rather than by peripheral rhythms or other behaviors.

The PVN is a common nuclei downstream of both the SCN and the ARC. Dissimilar to the ARC however, rhythmic release of GABA from the SCN, rather than α-MSH, onto parasympathetic PVN neurons regulates circadian fluctuations of glucose metabolism (Kalsbeek et al., 2011). Indeed, via the SCN, all three key end organs (e.g., pancreas, liver, muscle) exhibit daily changes in glucose regulation.

Within a healthy individual, plasma glucose exhibits a clear circadian rhythm with a rise just before the beginning of the active phase (Stenvers et al., 2012). Glucose tolerance also varies over of 24 h period. Notably, the glucose response to a standardized meal is substantially lower in the morning compared to the afternoon or evening (Van Cauter et al., 1992; Biston et al., 1996). In humans, but not nocturnal rodents, plasma insulin levels display a circadian rhythm with more pancreatic insulin production occurring in the morning than the evening (Baker and Jarrett, 1972; Carroll and Nestel, 1973; Sensi et al., 1973). This rhythm is reversed in nocturnal rodents. Skeletal muscle glucose uptake is highest in the morning via both insulin dependent (Verrillo et al., 1989) and insulin independent (Feneberg and Lemmer, 2004) mechanisms. While all of these changes would seem to explain the improved glucose tolerance, HGP also displays its own circadian rhythm. Indeed, HGP rises during the active period and decreases during sleep (Clore et al., 1989). Thus, at the start of the active period in humans, HGP is comparatively low while insulin production and skeletal muscle glucose uptake are high. Together these changes can account for the improved glucose tolerance to a morning meal. Importantly, SCN induced changes in HGP occur independently of insulin, glucagon, meal timing, and local liver clocks (Cailotto et al., 2005). The SCN maintains these daily rhythms (la Fleur et al., 2001) and changes in glucoregulation via innervation of the PVN and the ANS (Kalsbeek et al., 2004; Cailotto et al., 2008). Within the SCN, pre-parasympathetic and pre-sympathetic neurons (Buijs et al., 2003), synapse onto the PVN which result in insulin release from the pancreas (Kalsbeek et al., 2011) and glucose production in the liver (Kalsbeek et al., 2004), respectively. Importantly, the balance between the autonomic system and the rhythms of glucose production and glucose uptake across the active and rest phases results in steady plasma glucose levels and a shifting fuel to the peripheral tissues when it is needed.

Furthermore, the SCN may also control glucose metabolism through two-way communication with the ARC. Indeed, the SCN synapses onto both the POMC and NPY/AgRP expressing neurons in the ARC (Froy, 2010). The SCN itself also expresses receptors for both leptin and ghrelin (Guan et al., 1997; Saper et al., 2005; Zigman et al., 2006) raising the possibility that these hormones may directly regulate the SCN in a manner similar to their regulation on the ARC. Furthermore, NPY from the intergeniculate leaflet (Harrington, 1997) and 5HT from the raphe nuclei have been shown to modulate SCN signaling (Challet, 2007). While it is unknown if nutrient signals can act within the SCN, changes in AMPK activity can phosphorylate the core circadian gene, cryptochrome 1 (*Cry1*) and lead to a lengthening of endogenous circadian period within peripheral tissues (Lamia et al., 2009). It is important to point out that as metabolic pathways change with obesity (e.g., rising leptin levels), these changes can feedback to the SCN and lead to rhythm changes. These rhythm changes, in turn, could result in circadian disruption.

An increasing body of literature highlights the link between circadian disruption and disease including cardiovascular disease, Alzheimer’s, cancer, reproductive dysfunction, and important for this review, T2DM (Tenkanen et al., 1998; Morikawa et al., 2005; Boivin et al., 2007; Haupt et al., 2008; Rüger and Scheer, 2009; Wang et al., 2011). Many of the metabolic links to circadian disruption have not yet defined clear mechanisms of action but several correlational studies have been performed. For example, night-shift workers exhibit shifted glucose and insulin rhythms (Simon et al., 2000) and have a moderately higher risk of developing T2DM compared to their day-shift equivalents (Wang et al., 2011). In addition, eating during the wrong time of day has been linked to weight gain and impaired glucose metabolism (Bazotte et al., 1989; Colles et al., 2007; Salgado-Delgado et al., 2008; Arble et al., 2009; Fonken et al., 2010). The use of genetic models has greatly progressed our mechanistic understanding of the link between circadian rhythms and metabolism. One of the first circadian and metabolic models was that of the *Clock* mutant mouse, which has a mutation in a core circadian gene, *Clock*. This mouse displays elevated body weight, fat mass, glucose, leptin, and lipid profiles (Turek et al., 2005). Mutation of other core circadian genes has also been linked to glucose metabolism. Mutation of the gene, brain, and muscle aryl hydrocarbon receptor nuclear translocator (ARNT-like 1 or BMAL1), only within pancreatic β-cells results in decreased plasma insulin levels, elevated fasting glucose levels, and an overall imbalance to glucose metabolism (Marcheva et al., 2010). Whereas knockout of BMAL1 specifically in the liver leads to hypoglycemia (Lamia et al., 2008). Knockout of the core circadian genes *Cry1* and *Cry2* results in elevated glucose and glucose intolerance (Zhang et al., 2010). Interestingly, over-expression of *Cry* improves glucose tolerance in otherwise insulin resist and glucose intolerant *db/db* mice (Zhang et al., 2010). These studies highlight the interconnected pathways between the circadian and metabolic system at the cellular and genetic level.

Taken together, the circadian system has a prominent effect on glucose metabolism. Working primarily through the ANS to generate daily rhythms in glucose parameters, the SCN, and circadian rhythms are also integrated to metabolic pathways at the brain, tissue, cellular, and genetic level.

### Implication for treatment

It is important to consider that many humans who experience circadian disruption due to shift work or altered sleep patterns, also consume high fat diets. While each of these challenges alone are known to affect glucose metabolism, it is possible that the combined effect of the two challenges are larger and more detrimental to glucose and/or overall health than the sum of each single factor. For example, merely consuming a high fat diet is known to alter the endogenous circadian clock resulting in a lengthened circadian rhythm and a delay in the expression of clock and clock controlled genes (Kohsaka et al., 2007). Similarly, some studies indicate that circadian disruption can increase fat consumption (Lowden et al., 2010). Thus, a feed-forward loop between high fat diets and circadian disruption could work in an additive manner to propagate metabolic disease. For effective treatment, knowledge of not only the single isolated stressor, but also the interactive properties of that stressor would be ideal. By specifically targeting the source of glucose homeostasis, CNS treatment could present novel and more efficacious treatment options.

A major limitation to targeting the CNS for treatment of obesity and/or T2DM is that in some signaling systems, CNS action may have opposite downstream metabolic effects. Two T2DM therapeutics have this problem: insulin, and glibenclamide. Although CNS insulin action is catabolic, T2DM patients gain weight as a result of insulin treatment. This likely results from the balance of the anorexigenic action of the hormone within the CNS (Woods et al., 1979) versus its potent stimulatory effect on fatty acid synthesis in the periphery (Porte et al., 2005). Glibenclamide, which inhibits K_ATP_ channels and results in an increase in insulin secretion, acts centrally to cause hepatic insulin resistance (Pocai et al., 2005a). The fact that this drug is an effective hypoglycemic agent suggests the peripheral action sufficiently counteracts its actions within the CNS. One last class of therapeutics worthy of mention is thiazolidinediones which function as insulin sensitizers. The problem with thiazolidinediones is slightly different in that its central and peripheral actions are the same and function to both improve glucose homeostasis (Lu et al., 2011), however, they also function to increase adiposity (Lu et al., 2011; Ryan et al., 2011). To counteract the undesirable effects of a single treatment, one option is to use a combination of therapeutics that would be more effective at treating obesity. Another form of combined therapeutics that is being increasingly explored is designed to target both obesity and glucose homeostasis.

Interestingly, bariatric surgery is one form of combined therapy for both obesity and T2DM. Bariatric surgery has long been linked with sustained weight loss and recent research highlights the ability of bariatric surgery to act as an independent treatment of metabolic disease including T2DM (Cummings, 2012). Indeed, bariatric surgeries such as RYGB and VSG are now being referred to as “metabolic surgeries” in reference to their efficiency in alleviating T2DM quickly and before substantial weight loss. It is hypothesized that such metabolic surgeries may directly affect the brain via the gut-brain axis that in turn can influence HGP. For example, duodenal-jejunal bypass surgery has been shown to lower glucose concentration by increasing nutrient flow across the nutrient sensing jejunum. Indeed, this increased nutrient signaling was shown to lead to a decrease in glucose via it action on the CNS (Breen et al., 2012). Bariatric surgery also leads to increases in postprandial CCK and GLP-1 which, as described previously, can regulate glucose metabolism (Rhee et al., 2012). Moreover, there is some research, although limited, which links bariatric surgery to circadian organization (Colles and Dixon, 2006; Michalaki et al., 2009; Stearns et al., 2009), further suggesting the gut-brain connection and how it can also influence the SCN.

However, the costs and invasiveness of bariatric surgeries prevents wide implementation and thus, additional treatment options are necessary. In principle, as circadian disruption has now been extensively linked to metabolic dysfunction, future treatment advances could use combined therapies that target both circadian disruption and obesity. Chronotherapy, which traditionally refers to utilizing the time of administration to optimize treatment, can now refer to targeting the circadian system as a form of treatment. Melatonin, for example, plays a key role in regulating circadian rhythms and also positively effects metabolism by increasing triglyceride clearance (Garaulet and Madrid, 2010). Additionally, quick release bromocriptine, a dopamine 2 receptor agonist, has recently been approved by the FDA for treatment of T2DM. Interestingly, bromocriptine is only effective if taken in the morning upon awakening and is hypothesized to work via the circadian system to substantially reduce and regulate daily glucose levels in T2DM (Kerr et al., 2010).

Thus, the CNS is clearly important in regulating glucose homeostasis under various physiological challenges including stress, meals, and circadian variations. Further, environmental pressures such as high fat diets and circadian disruptions alter the ability of the CNS orchestrate the peripheral organs to maintain glucose levels. We hypothesize that this contributes to the onset of T2DM. An increased understanding of how glucose metabolism responds to environmental challenges can lead to new treatments. Indeed, strides are currently being made to develop combination treatments that target both the cause and the symptoms of impaired glucose metabolism.

## Conflict of Interest Statement

The authors declare that the research was conducted in the absence of any commercial or financial relationships that could be construed as a potential conflict of interest.
